# Quantitative longitudinal investigation of non-alcoholic steatohepatitis in mice by photoacoustic microscopy

**DOI:** 10.1016/j.pacs.2025.100741

**Published:** 2025-06-11

**Authors:** Jianshuang Wei, Ren Zhang, Mingchen Jiang, Lulu Gao, Ximiao Yu, XiuLi Liu, Yanfeng Dai, Qingming Luo, Zhihong Zhang, Xiaoquan Yang

**Affiliations:** a MOE Key Laboratory for Biomedical Photonics, Wuhan National Laboratory for Optoelectronics, Huazhong University of Science and Technology, Wuhan 430074, China; b State Key Laboratory of Digital Medical Engineering, Key Laboratory of Biomedical Engineering of Hainan Province, School of Biomedical Engineering, Hainan University, Haikou 570228, China

**Keywords:** Photoacoustic microscopy, Non-alcoholic steatohepatitis, Longitudinal investigation, Three-dimensional quantification

## Abstract

Non-alcoholic steatohepatitis (NASH) is a prevalent chronic liver disease characterized by significant alterations in liver microvascular structures, leading to microcirculatory dysfunction and potentially contributing to various extrahepatic complications. In this study, we propose a longitudinal investigative pipeline based on liver photoacoustic microscopy (LPAM), integrating optical-resolution photoacoustic microscopy (OR-PAM), a modular liver window (MLW), a custom 3D-printed liver imaging mount (LIM), and a dedicated vessel-sinusoid separation and analysis method. This pipeline enabled continuous monitoring and quantitative assessment of microvascular changes in a NASH mouse model over a six-week period. As NASH progressed, vessel density decreased by 64.18 %, and hepatic sinusoid vessel coverage was reduced by 77.38 %. Furthermore, hepatic sinusoidal volume, length, radius, tortuosity, and density declined by 87.29 %, 83.92 %, 21.86 %, 71.57 %, and 86.81 %, Analysis of hepatic sinusoidal branches revealed a 51.80 % decrease in the fractal dimension of composite branches and a 54.90 % increase in that of dead-end branches. These findings suggest that lipid accumulation and inflammatory responses contribute to the progressive deterioration of hepatic microvascular structures, thereby exacerbating vascular damage. LPAM offers a high-resolution, label-free imaging approach for dynamic monitoring of NASH-associated microvascular alterations. This study advances our understanding of hepatic microcirculatory changes in NASH and provides valuable insights for both basic research and clinical management.

## 1 Introduction

Non-alcoholic steatohepatitis (NASH) is a progressive subtype of non-alcoholic fatty liver disease (NAFLD) [1]. Currently, NAFLD affects approximately 25 % of the global population [2], and with the rising incidence of metabolic syndrome, the prevalence of NASH is expected to increase. The liver microvascular system plays a vital role in maintaining liver function by delivering essential nutrients and oxygen to hepatocytes. During NASH progression, structural abnormalities and distortions in the microvascular architecture are among its hallmark features. These alterations begin with the narrowing of sinusoidal spaces due to lipid accumulation in hepatocytes and are further exacerbated by impaired blood flow resulting from collagen deposition within the sinusoidal lumen. Such structural disruptions lead to microcirculatory dysfunction, compromising oxygen delivery and metabolite exchange, which may eventually result in complications such as portal vein thrombosis [3] and atherosclerosis [4].

Given the progressive nature of NASH-related microvascular damage, long-term dynamic monitoring is essential to fully understand disease progression. However, conventional visual inspection of microvascular images, which relies on the researcher’s subjective judgment, is insufficient for accurately capturing and quantifying these dynamic changes. Moreover, advances in medical imaging technologies have made microvascular images increasingly information-rich and structurally complex, further limiting the effectiveness of visual assessment. Therefore, developing quantitative analysis methods based on three-dimensional (3D) vascular imaging is critical. Such methods allow for objective evaluation of microvascular structural changes and enable the identification of NASH progression patterns through long-term tracking, thereby providing more reliable evidence for clinical diagnosis and treatment.

Traditionally, histological sectioning [5] has been the primary approach for quantitatively analyzing vascular morphology, including parameters such as vessel density, length, and diameter. However, this method requires staining and microscopic observation of tissue slices only a few micrometers thick and within a limited field of view. In addition, the sectioning process can damage vascular structures, potentially compromising measurement accuracy. By contrast, imaging modalities such as Magnetic Resonance Imaging (MRI), Ultrasound (US), Positron Emission Tomography (PET), and optical imaging provide more convenient methods for acquiring 3D vascular data and tracking dynamic changes in liver vasculature during NASH. Despite their advantages, these techniques have notable limitations: MRI is time-consuming, and unsuitable for patients with certain implants [6]; PET involves radiation exposure [7]; and although US is safe and widely accessible [8], it lacks the resolution needed for detailed microvascular imaging. Optical imaging [9], while capable of high-resolution imaging, typically requires tissue clearing to increase penetration depth, making it unsuitable for real-time, in vivo monitoring.

In contrast to these traditional non-invasive techniques, photoacoustic tomography (PAT) utilizes hemoglobin as an endogenous contrast agent [10], [11], [12], [13], enabling high-sensitivity, high-resolution imaging of deep vascular structures. Among PAT techniques, optical-resolution photoacoustic microscopy (OR-PAM) offers optical resolution in the range of 1–10 μm [14], allowing for 3D visualization of the microvascular network in the livers of live small animals. This makes OR-PAM particularly well-suited for investigating structural changes in liver microvasculature associated with NASH. As an emerging non-invasive imaging modality, OR-PAM holds significant potential in microscopic imaging and represents a valuable tool for studying microvascular alterations during NASH progression.

OR-PAM has been widely applied in liver research [15], [16], [17], [18], yet two significant challenges remain. First, earlier studies frequently relied on invasive techniques, such as abdominal dissection, full liver exposure, and organ fixation during imaging. These procedures cause considerable harm to mice and are unsuitable for long-term monitoring in NASH studies. To address this limitation, researchers combined PAM with a drawer-type liver window technique to observe dynamic changes in liver microvasculature in a B16 liver metastasis mouse model [19]. Although this method significantly reduces image interference caused by physiological movements such as respiration and heartbeat, the liver, a soft organ dependent on physiological motion and situated near other abdominal structures, remains susceptible to mechanical influences. These include diaphragm contractions, gastrointestinal peristalsis, and blood pulsation. Such multidimensional motion artifacts can cause subtle liver displacements within the imaging window, leading to residual motion blur that compromises image stability and accuracy, especially when tracking dynamic changes in individual microvessels.

Second, the liver microvasculature consists of hepatic sinusoids and vessels with intact vascular walls, such as central veins and portal vein branches. However, most existing analytical methods fail to distinguish between these structures, relying instead on image-wide quantitative analysis. This often results in signal overlap and difficulties in independently extracting key parameters. For instance, vessel diameter measurements may be skewed by overlapping signals, hindering accurate distinction between sinusoids and larger vessels. Blood volume and density estimations can also be compromised by signal interference, reducing measurement reliability. Moreover, critical structural features (such as tortuosity and fractal dimension) are difficult to assess precisely in the early stages of disease progression using current approaches.

Therefore, this study proposes the Liver Photoacoustic Microscopy (LPAM) technique for long-term monitoring of structural changes in liver microvasculature during NASH progression. LPAM enables the separation of hepatic sinusoids from larger vessels and supports the extraction and analysis of quantitative parameters from 3D data. To validate the effectiveness of LPAM, comparative experiments were first conducted to demonstrate improvements in imaging quality during long-term monitoring. Subsequently, in vitro simulation experiments using microtubes and lipid emulsions were designed to assess the impact of lipid accumulation on photoacoustic (PA) signals within liver microvasculature. Finally, a quantitative evaluation of the proposed vessels-sinusoids separation method was performed, and its utility in NASH-related research was validated.

## 2 Materials and methods

### 2.1 Long-term in vivo imaging system for mouse liver

**Fig. 1:**
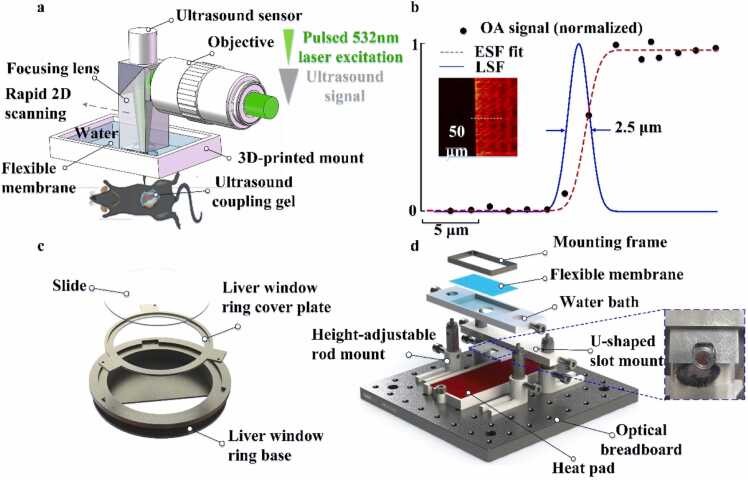
Schematic of the imaging setup - longitudinal monitoring of NASH disease model mice using WF-LPAM and a 3D-Printed bracket integrated with LWM: (a) a 532 nm short-pulse laser is focused onto the mouse liver via an ultrasound coupling prism, generating ultrasonic waves through localized light absorption. A sensor scans a fixed liver volume to produce high-resolution images. (b) Resolution characterization using a resolution board, showing lateral resolution of 2.5 µm measured by the full width at half maximum (FWHM) of the line spread function (LSF). black dots indicate measured PA signals; the red dashed line represents the edge spread function (ESF) fitted by a logistic model; the blue solid line corresponds to the numerical derivative of the ESF, representing the LSF. (c) Photograph of the MLW used for imaging. (d) Image of the custom 3D-printed LIM bracket.

The imaging system (Fig. 1(a)) uses a laser pulse with a wavelength of 532 nm, a pulse width of 9 ns, and a pulse repetition rate of 1 kHz [20], [21]. The laser beam is delivered through a single-mode optical fiber (core diameter: 3.5 μm) and focused onto the sample using an objective lens. When the laser pulses are absorbed locally by blood vessels, they generate broadband ultrasonic signals, which are detected by a coaxially aligned wideband ultrasound transducer. By employing a single-mode optical fiber and a low numerical aperture objective (NA = 0.05), the system achieves a lateral resolution of 2.5 µm (Fig. 1(b)) and an imaging depth of approximately 1 mm.

To enable longitudinal imaging of the same mouse over time, a specialized imaging platform was developed, consisting of two main components: a modular liver window (MLW) and a custom 3D-printed liver imaging mount (LIM). The MLW consists of two parts: a liver window ring base and a ring cover plate, as shown in Fig. 1(c). The imaging window slide is affixed to the cover plate and integrated with the ring base via a rotational mechanism, forming a modular imaging window for liver observation in mice. The design is inspired by abdominal imaging windows used in optical microscopy. To improve biocompatibility and durability, the liver window ring base material was upgraded from acrylic to titanium, and the earlier multi-part tray-base structure was simplified into an integrated design. The detachable cover plate allows for the adjustment of the relative positions of internal organs (e.g., liver, spleen, and small intestine), helping ensure minimal deviation in imaging regions across sessions. To enhance signal penetration, the abdominal window material was changed from borosilicate or resin/acrylic glass to ultra-thin polymethyl methacrylate (Ut-PMMA), improving PA transmission efficiency.

The LIM includes a movable imaging window and a movable U-shaped clamp, mounted directly above the animal bed (Fig. 1(d)). The setup is built on a 15 cm × 15 cm optical breadboard (MSB15/M, Thorlabs), which supports metal rods that secure both the animal bed and the framework for the imaging window and clamp. A custom adjustable heating pad is placed on the bed to maintain body temperature during anesthesia. It includes a headrest with a built-in control panel for real-time temperature monitoring and regulation. The imaging window itself comprises two sections: the upper layer houses the components for light delivery and PA signal collection through a transparent polyethylene film, while the lower layer enables acoustic coupling between the liver tissue and the ultrasound transducer via a water bath. This bath includes a concave trough filled with water to accommodate the free movement of the imaging probe.

The U-shaped clamp, located at the bottom of the water bath, reinforces the MLW and is a core component for stabilizing liver imaging. By securely fixing the liver window ring in multiple dimensions, the clamp effectively suppresses breathing-induced motion artifacts. Both the imaging window and U-shaped clamp are supported by height-adjustable metal rods, allowing for flexible positioning relative to the mouse. This ensures optimal alignment between the U-shaped clamp, imaging window, and liver window ring for efficient PA excitation and signal transmission. The design and assembly of the MLW and LIM were completed using SolidWorks 2020.

### 2.2 Separation of vessels and hepatic sinusoids structures

**Fig. 2:**
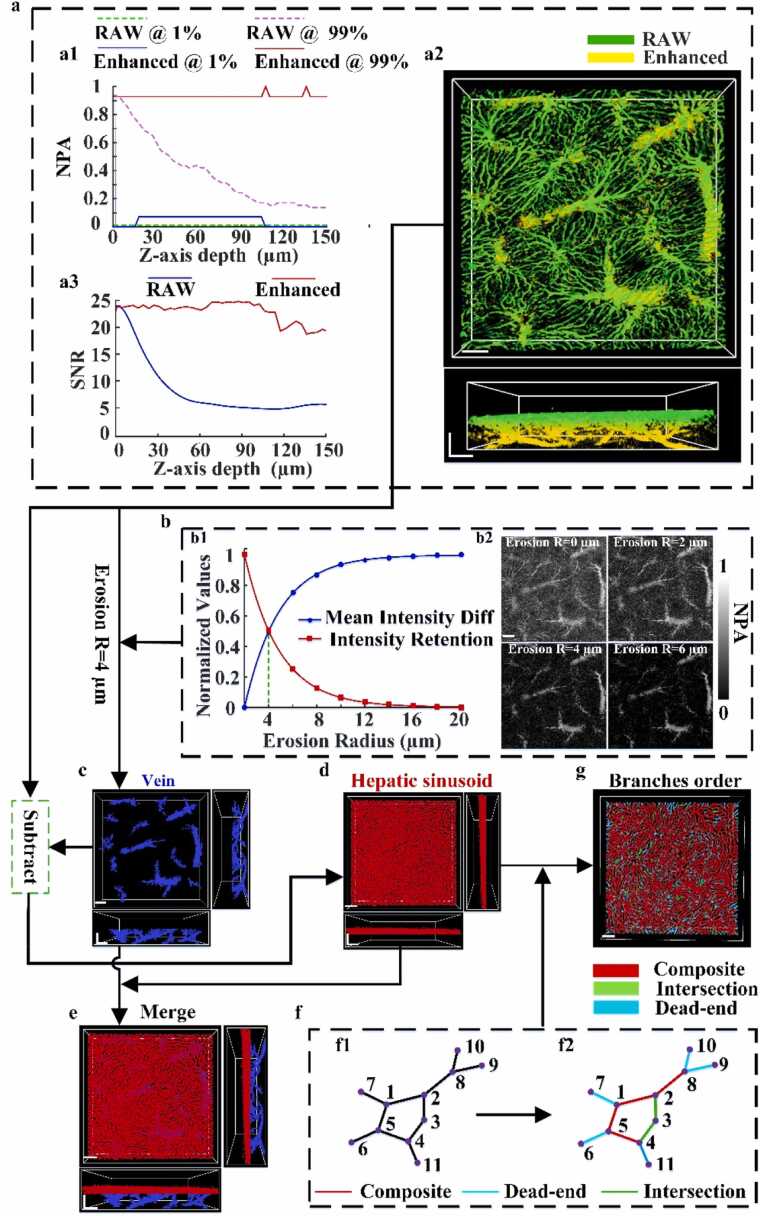
Schematic diagram of liver microvasculature processing method: (a) image enhancement based on percentile analysis and histogram matching. (b) Schematic of different corrosion tube diameter curves and corresponding images. (c) 3D binary image of separated vessels. (d) 3D binary image of separated hepatic sinusoids. (e) 3D fusion image of post-sinusoidal vessels. (f) Classification of hepatic sinusoid branches. (g) 3D image of classified hepatic sinusoid branches. Scale bar: 100 µm.

MATLAB software (R2021a, MathWorks) was used to separated vessels and hepatic sinusoids vessels structures, while Amira 3D software was employed for the 3D reconstruction of PA images. The overall workflow of the proposed method is illustrated in Fig. 2.

To mitigate depth-related signal degradation, an image enhancement method based on percentile analysis and histogram matching was used [22]. Specifically, a statistical analysis of pixel intensity was performed for each 2D slice along the Z-axis, extracting the 1st percentile (*I1*) and 99th percentile (*I99*) signal values. The difference between *I99* and *I1* was used to evaluate the intensity dynamic range of each slice. The slice with the largest intensity range was selected as the reference layer. The grayscale histogram of this reference slice was then used as a template, and histogram matching was performed on all remaining slices to align their intensity distributions with the reference. This process enhanced the contrast of both shallow and deep layers, improving inter-slice consistency and structural visibility across depths.

Fig. 2(a1) illustrates the variation in PA signal intensity across depths before and after enhancement. The green and pink dashed lines represent the 1st and 99th percentile values of the unenhanced images, while the blue and red solid lines show the corresponding values after enhancement. Fig. 2(a2) displays the 3D visualization results, with the green channel indicating the original image and the yellow channel showing the enhanced image. Fig. 2(a3) further presents the signal-to-noise ratio (SNR) as a function of depth, confirming that the enhancement not only significantly improves SNR but also maintains stability across the entire Z-axis.

After enhancement, morphological erosion was applied using spherical structural elements to approximately eliminate liver sinusoid signals. Previous studies have shown that due to variations in imaging systems and biological differences among individuals, the diameter of liver sinusoids can vary; however, it typically ranges between 3 and 5 μm [23], [24], [25], [26]. Therefore, the sinusoid diameter was used as a reference to set the radius of the structural element for erosion within this range. To determine the optimal erosion radius, the following approach was implemented: for each image processed with different erosion radii, two quantitative metrics, mean intensity difference (MID) and intensity retention (IR), were calculated. By analyzing and comparing these metrics, a radius was identified that effectively suppresses liver sinusoid signals while preserving vessels structures, thereby improving the accuracy of subsequent analysis. The formulas used to compute these metrics are presented in (1), (2):

(1) $$I_{\mathrm{MID}}=|{\overset{¯}{I}}_{c}-\overset{¯}{I}|$$

(2) $$R=\frac{\sum I_{c}}{\sum I}$$

where $\bar{I_{c}}$ represents the average intensity of the image after corrosion; $\bar{I}$ denotes the average intensity of the original image; $I_{\mathrm{MID}}$ reflects the impact of removing liver sinusoids during the corrosion process on the overall intensity of the image;$\sum I_{c}$ is the total intensity of all pixels in the eroded image; ∑I is the total intensity of all pixels in the original image; R is the IR value, which evaluates whether corrosion causes excessive loss of the overall intensity information of the vessels.

In Fig. 2(b1), the blue and red curves illustrate the normalized trends of MID and IR, respectively, as functions of the erosion radius. As the radius increases, MID gradually rises, while IR decreases sharply. The intersection of these two curves occurs at approximately 4 μm (marked by the green dashed line), where both metrics approach a value of 0.5. This point represents an optimal trade-off between enhancing intensity contrast and preserving image information. At this radius, the erosion process begins to effectively suppress hepatic sinusoid signals without significantly compromising structural content, and is thus selected as the optimal erosion radius. Fig. 2(b2) shows the results of erosion at various radii. Based on this analysis, morphological erosion was applied to Fig. 2(a2) to suppress hepatic sinusoid signals. Following erosion, an adaptive threshold segmentation algorithm [27] was employed, which calculates a local threshold based on the mean intensity within a sliding window. This method performs binarization to generate a preliminary binary image of vessels structures. Given that connected vessels components exhibit significantly larger volumes than residual sinusoids, the median volume of all connected components was used as a robust threshold to remove small, unrefined sinusoidal structures that were not adequately suppressed by morphological erosion. The final binary image of separated vessels is shown in Fig. 2(c).

**Fig. 3:**
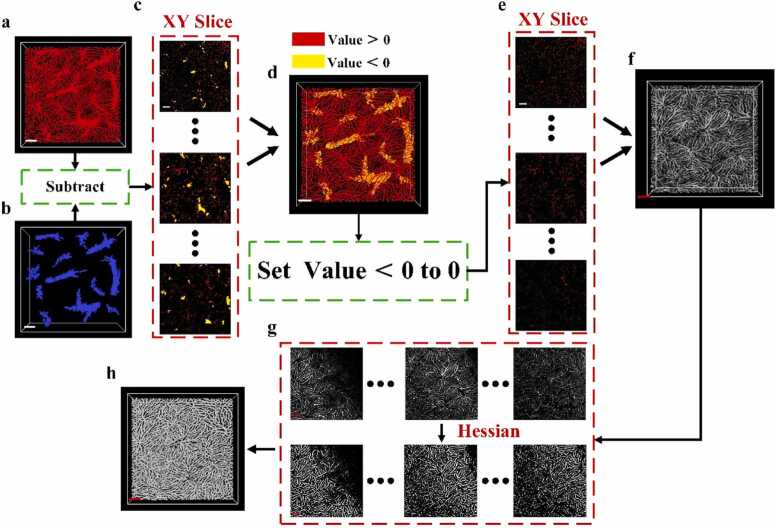
Schematic diagram of hepatic sinusoid separation method: (a) Contrast-enhanced image. Scale bar: 100 µm. (b) 3D binary image of separated vessels. (c) Xy cross-section of difference image after pixel subtraction; yellow represents pixels < 0, red represents pixels > 0. (d) 3D reconstruction of the difference image. (e) Xy cross-section of difference image after removing pixels with values < 0. (f) 3D reconstruction of (e). (g) 3D image of the separated hepatic sinusoids. (h) Xy cross-section after hessian enhancement. (I) 3D image after hessian enhancement. Scale bar: 100 µm.

Subsequently, separation of hepatic sinusoids was carried out, as illustrated in Fig. 3. First, the original image (Fig. 3(a), corresponding to Fig. 2(a2)) was subtracted from the binary vessels separation image (Fig. 3(b), corresponding to Fig. 2(c)) using a pixel-wise operation. This yielded a different image, with its XY cross-section shown in Fig. 3(c). In the difference image, yellow regions represent areas where vessels pixels were subtracted from the original image, resulting in negative values. Red regions indicate areas corresponding to hepatic sinusoids in the original image, which retain positive values after subtraction. The 3D reconstruction of the difference image is shown in Fig. 3(d). To isolate the hepatic sinusoids, all negative values in the difference image were set to zero, retaining only the positive values associated with sinusoid structures. The resulting XY cross-section and 3D reconstruction are displayed in Fig. 3(e) and Fig. 3(f), respectively. To further enhance the tubular features of hepatic sinusoids, a 3D Hessian matrix-based filtering method was applied, which accentuates tubular structures. The resulting XY cross-section is shown in Fig. 3(g), and the corresponding 2D reconstruction is presented in Fig. 3(h).

Finally, an adaptive segmentation algorithm was applied to the enhanced image, producing the final binary image of separated hepatic sinusoids shown in Fig. 2(d). When the separated vessels and hepatic sinusoids are merged (Fig. 2(e)), the combined visualization clearly illustrates the structure and spatial distribution of both hepatic sinusoids and vessels. The separation of vessels and hepatic sinusoids provides a robust foundation for subsequent morphological studies and quantitative analyses of the liver microvasculature.

### 2.3 Statistical analysis

After completing the separation of both hepatic sinusoids and vessels, the next step involved measuring and comparing structural parameter changes of the vasculature across four distinct stages of NASH. Statistical analysis was performed using OriginPro 2021 software (OriginLab, USA). Mann-Whitney U tests were employed to compare results between different stage groups, with a two-sided *P* value less than 0.05 considered statistically significant. Quantitative data related to liver microvasculature parameters are presented as mean ± standard deviation.

Several quantitative parameters were extracted to comprehensively characterize hepatic microvascular structure and function. Radius, volume, length, and tortuosity were measured to describe the fundamental structural features of the sinusoids network and to evaluate pathological changes in microcirculation. Density, vessel number, and fractal dimension (FD) were analyzed to reflect the complexity, spatial distribution, and dynamic properties of the vessels network. Additionally, the vessel coverage ratio was calculated to assess the spatial relationship between sinusoids and vessels, providing insight into the structural integrity and connectivity of the microvascular architecture. Table 1 lists the various parameters requiring quantification along with their respective quantification purposes.

**Table 1:** Quantitative parameters of hepatic microvasculature.

**Category**	**Parameters**	**Purpose**
Structural features	Radius\Volume\Length\Tortuosity	Pathological changes in microvascular
Distribution features	Density\Vessel number\FD	Dynamic characteristics of microvascular
Spatial features	Vessel coverage ratio	Integrity and connectivity of microvascular

The analysis was initiated by focusing on the hepatic sinusoids. First, a 3D thinning algorithm [28] was used to obtain a skeletonized representation of the separated hepatic sinusoids, which was subsequently transformed into a graph structure. This graph structure enabled the extraction of morphological parameters, including length, volume, average radius, and average tortuosity of the hepatic sinusoids. The length was defined by the total number of skeleton pixels, while the volume was calculated by counting the total number of foreground pixels in the 3D binary image of separated hepatic sinusoids. Modeling the microvasculature as cylinders, the radius was derived from the relationship between the cylinder’s volume and length. The tortuosity was defined as the ratio of the Euclidean distance between the first and last nodes of the skeleton to the total length.

Next, the distribution density of the hepatic sinusoids (*D*) was calculated, defined as the ratio of the hepatic sinusoid volume (*V*_*S*_) to the remaining volume after subtracting the vessel volume (*V*_*L*_) from the total volume (*V*), as shown in Eq. (3):

(3) $$D=\frac{V_{S}}{\left(V-V_{L}\right)}$$

This definition more precisely describes the distribution of the hepatic sinusoids in the non-vascular regions, thereby avoiding interference from changes in vascular structures in the calculation of the distribution density.

Based on the skeleton graph, hepatic sinusoid branches were classified into three types [26] (Fig. 2(f)): dead-end (cyan), crossing (green), and complex (red). Dead-end branches represent terminal paths (e.g., nodes 8–10, 4–11), often indicating obstructed flow. Crossing branches (e.g., nodes 3–4, 2–3) connect adjacent nodes and reflect network connectivity. Complex branches (e.g., nodes 1–5, 1–2) consist of multiple segments and act as major conduits for blood flow. This classification was performed globally across the entire 3D skeleton, ensuring spatial continuity and preserving the full topological structure. This approach enables quantification of microvascular variations and offers valuable insights into tissue microcirculation. The final result is illustrated in Fig. 2(g).

Next, the rate of change in FD for each branch type between the healthy and disease groups over different time points was statistically analyzed. The FD of blood vessels quantitatively reflects the complexity of the vascular network, with higher values typically indicating increased complexity. In this study, the box-counting method was applied, in which the 3D space occupied by the vessels is divided into cubes of side length *L*, and the number of cubes *N*(*L*) required to fully cover the vascular structure is counted. As *L* decreases, the FD is calculated by the following limit:

(4) $$\mathrm{FD}=-\underset{L\to 0}{\lim}\frac{\log N\left(L\right)}{\log L}$$

**Fig. 4:**
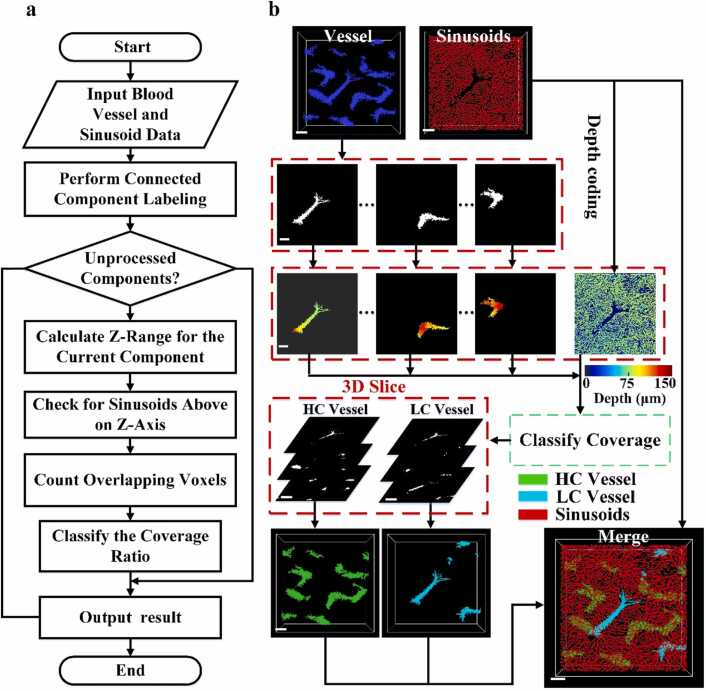
Processing method for spatial relationship between hepatic sinusoids and vessels: (a) flowchart of method. (b) Illustrative diagram corresponding to the method described in (a).

Subsequently, quantitative metrics for vessels were focused on, specifically tracking changes in vessel numbers and vascular coverage across various stages of disease progression. To quantitatively assess the coverage of hepatic sinusoids over vessels in different stages of the disease model, a method was devised to quantify this coverage. The methodology is outlined in Fig. 4, where Fig. 4(a) presents the workflow, and Fig. 4(b) provides a corresponding schematic illustration.

Next, based on the 3D spatial position of each vessel *V*_*i*_(*x*, *y*, *z*), the maximum index value Z_*max*_ along the Z-axis was extracted, representing the deepest layer containing the vascular signal. Here, *V*_*i*_(*x*, *y*, *z*) denotes the binary mask of the *i-th* vessel.

(5) $$Z_{\max}=\left\{z\left|\;\exists \right)(x,y),V_{i}(x,y,z)=1\right\}$$

where *z* denotes the layer index along the Z-axis (depth information) in the image and (*x*, *y*) represents the planar coordinate values at a specific layer z.

Simultaneously, in the binarized image *S*(*x*, *y*, *z*) of the hepatic sinusoids, the regions from the deepest layer of the vessel and below were set to zero, retaining only the uppermost part of the vessel *S'*(*x*, *y*, *z*). This step was designed to eliminate the hepatic sinusoid signals positioned beneath the vessel.

(6) $$S\prime (x,y,z)=\left\{\begin{array}{c} S(x,y,z),\text{ z}\leq Z_{\max}\\ 0,\text{ z}\leq Z_{\max} \end{array}\right)$$

Next, a bitwise AND operation was performed to overlay the vessel mask with the hepatic sinusoid mask on a slice-by-slice basis, producing a vessel mask that highlights regions overlapped by hepatic sinusoids. The number of overlapping voxels within this combined mask was then counted. The coverage rate$R^{i}$for each vessel was calculated as the ratio of overlapping voxels to the vessel’s total voxel count, according to the formula below. Based on their coverage rates, vessels were subsequently classified into high-coverage and low-coverage groups.

(7) $$R^{i}=\frac{\sum \left[V_{i}(x,y,z)\wedge S\prime (x,y,z)\right]}{\sum Vi(x,y,z)}$$

This method effectively distinguishes between vessels with high and low coverage rates, providing a precise and efficient way to quantify vascular coverage. It also visually illustrates the spatial relationship between vessels and hepatic sinusoids, delivering crucial quantitative insights for investigating microcirculatory alterations in disease models.

### 2.4 Simulation experiment and preparation of animals

In NASH, excessive lipid accumulation within hepatocytes forms large lipid vacuoles that occupy substantial intracellular space. These vacuoles compress normal cellular structures and can alter red blood cell distribution within the liver vasculature. To assess lipid effects on PA signals of hepatic microvasculature, an in vitro simulation experiment was conducted. Agarose gel served as a tissue-mimicking matrix, into which lipid emulsions at concentrations of 3 %, 5 %, and 7 % were uniformly mixed to model fatty liver conditions. Red microtubes, simulating blood vessels, were embedded at varying depths (0–1 mm) within both control (PBS) and lipid-containing agarose matrices

All animals were obtained from the Hubei Provincial Laboratory Animal Research Center. Animal studies were approved by the Hubei Province Committee on the Care and Use of Animals and conducted under the guidelines of the Animal Ethics Committee of Huazhong University of Science and Technology (Ethics Number: 844). Female C57BL/6 mice aged 6–8 weeks were housed under a 12-hour light/dark cycle. The NASH model was established using an MCD diet, feeding adult male mice 10 g of MCD feed per 20 g of body weight, with feed refreshed every two days for three weeks; water was provided ad libitum. The six-week observation was divided into four stages: normal (week 0), early (week 2), middle (week 4), and late (week 6). This allowed quantitative analysis of NASH progression impacts on mouse liver microvasculature through staged imaging.

## 3 Result

### 3.1 Performance of MLW & LIM

The PAM integrated with the MLW&LIM system enables longitudinal in vivo liver imaging in mice. This protocol features a liver imaging window that provides long-term tissue stability, allowing high-resolution data acquisition of liver microvascular structures for up to six weeks and enabling 3D reconstruction with a field of view (FOV) of up to 2 mm × 2 mm.

**Fig. 5:**
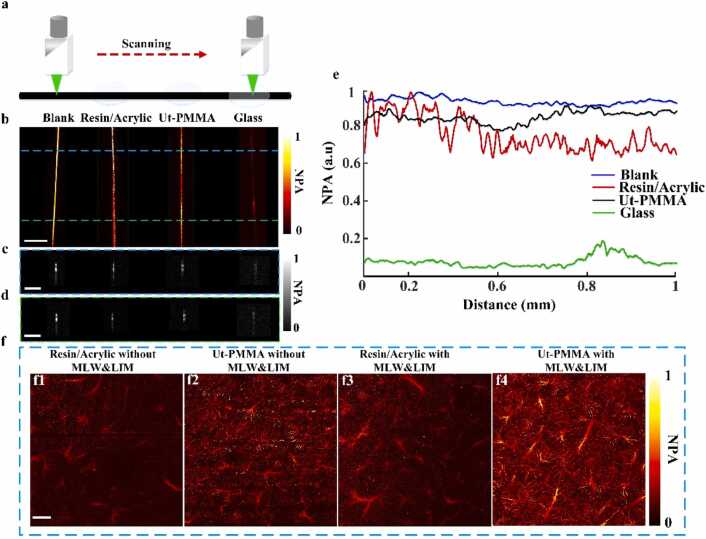
LPAM verification and in vitro simulation experiments: (a) scanning schematic of a 7 µm carbon fiber covered by control group, acrylic/resin, ultra-thin PMMA, and borosilicate glass. (b) Pa maximum amplitude projections (MAPs) and normalized PA amplitude (NPA). (c-d) Cross-sectional images on the XZ plane corresponding to the blue and Green dashed lines in (a), respectively. (e) Comparative curves of average PA signal across cross-sections as a function of distance along the Y-axis for the control group and three material types. (f) Liver PA MAPs acquired using different methods. Scale bar: 100 µm.

The MLW slide material was improved to enhance signal penetration by using Ut-PMMA. To demonstrate the superiority of Ut-PMMA, an experiment was conducted by placing a 7 µm carbon fiber filament beneath three materials, glass shards, resin/acrylic glass, and Ut-PMMA, followed by sequential imaging (schematic shown in Fig. 5(a)). Maximum amplitude projection (MAP) images for these materials are presented in Fig. 5(b), arranged left to right as Blank, resin/acrylic glass, Ut-PMMA, and glass shards. Corresponding XZ cross-sectional images along the blue and green dashed lines appear in Fig. 5(c) and Fig. 5(d), respectively.

The average PA signal in the XZ plane along the Y-axis was quantified, and normalized signal variations were plotted in Fig. 5(e). The horizontal axis represents the distance along the Y-axis, while the vertical axis indicates the normalized photoacoustic signal. The blue, red, black, and green curves correspond to the blank control, resin/acrylic glass, Ut-PMMA, and borosilicate glass, respectively. As shown in the graph, the glass shard showed the weakest signal, about 15 % of the blank, with minimal variation along the Y-axis. The resin/acrylic glass exhibited unstable amplitude fluctuations, dropping approximately 40 % from its peak, impairing reliable signal acquisition and quantitative analysis. In contrast, Ut-PMMA maintained a stable signal at roughly 80 % of the blank’s amplitude, demonstrating excellent PA transmission efficiency and optimal balance. Moreover, while adding resin to acrylic can improve localized signal acquisition, the point-scanning nature of PAM can cause rapid local temperature increases, making resin-containing acrylic more prone to deformation and signal instability compared to pure acrylic.

To validate these findings, livers from mice were imaged under four conditions (Fig. 5(f)): resin/acrylic without LIM (Fig. 5(f1)), resin/acrylic with LIM (Fig. 5(f2)), Ut-PMMA without LIM (Fig. 5(f3)), and Ut-PMMA with LIM (Fig. 5(f4)). The Ut-PMMA with LIM yielded the highest image quality, effectively eliminating motion artifacts and producing the strongest PA signals.

For quantitative image quality assessment, the Gray Level Co-occurrence Matrix (GLCM) method was employed [29]. GLCM-based texture features (i.e., contrast, entropy, and homogeneity) were calculated for each imaging method, combined into a composite score, and normalized relative to the highest score. Results showed that resin without LIM scored 0.09, resin with LIM 0.19, and Ut-PMMA without LIM 0.38, confirming the superior performance of Ut-PMMA with LIM.

The MLW&LIM system offers a rapid, cost-effective, and user-friendly solution with enhanced imaging stability. Using Ut-PMMA as the MLW slide material improves signal penetration and PA transmission efficiency. The LIM, fabricated entirely from standard 3D-printed components and biocompatible titanium alloy, ensures safety, reproducibility, adaptability, and ease of use.

### 3.2 Simulation experiment analysis

**Fig. 6:**
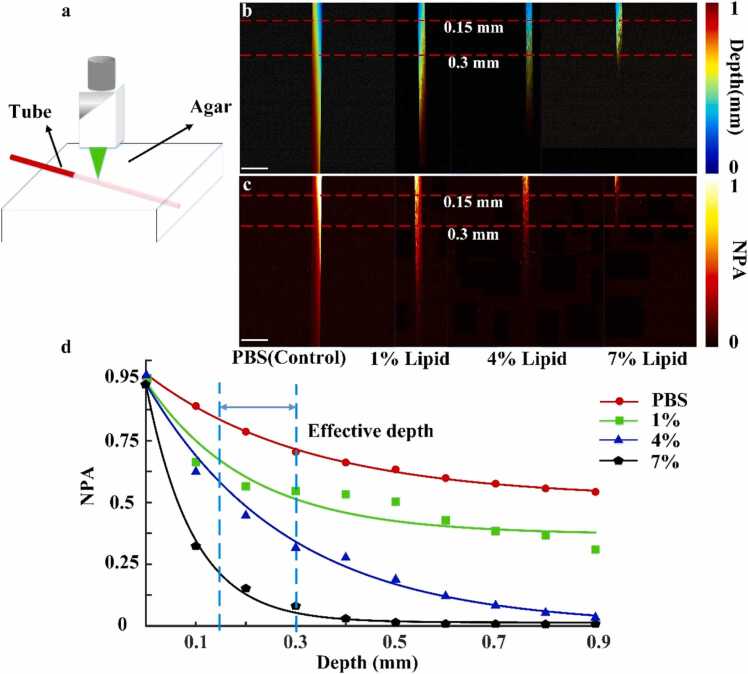
Simulation experiment results: (a) schematic of the simulation experiment. (b) Depth-coded PA images of microtubes in the control group and with varying lipid emulsion concentrations. (c) Maps of microtubes across the control and lipid emulsion groups. (d) Attenuation curves for the control group and different lipid emulsion concentrations. Scale bars: 1 mm.

The effective imaging depth for liver microvasculature was concentrated between 0.15 mm and 0.3 mm. To ensure consistency across experimental conditions, all microvascular analyses were confined to this depth range, as illustrated in the experimental schematic (Fig. 6(a)). Fig. 6(b) displays depth-coded PA images of microtubes under control and varying lipid emulsion concentrations, with depth information represented in pseudo-color (0–1 mm range). Fig. 6(c) presents the corresponding MAPs for each group. The two red dashed lines in Fig. 6(a) and Fig. 6(b) indicate the 0.15 mm and 0.3 mm depth boundaries.

Experimental results showed a positive correlation between lipid concentration and PA signal attenuation within the effective depth window. To quantify this effect, attenuation curves were plotted for each group (Fig. 6(d)), with depth on the x-axis and normalized PA signal intensity on the y-axis. The dashed lines in the figure delineate the effective depth window.

Within this window, PA signal intensities decreased by 34.23 %, 37.95 %, 48.75 %, and 76.67 % for the 1 %, 4 %, 7 % lipid groups, respectively, compared to the PBS control group. These findings indicate that increased lipid concentration leads to significantly greater PA signal attenuation. The results suggest a strong positive correlation between lipid accumulation and signal loss, implying that excessive lipid buildup can markedly impair PA imaging of hemoglobin.

### 3.3 Liver microvasculature separation

**Fig. 7:**
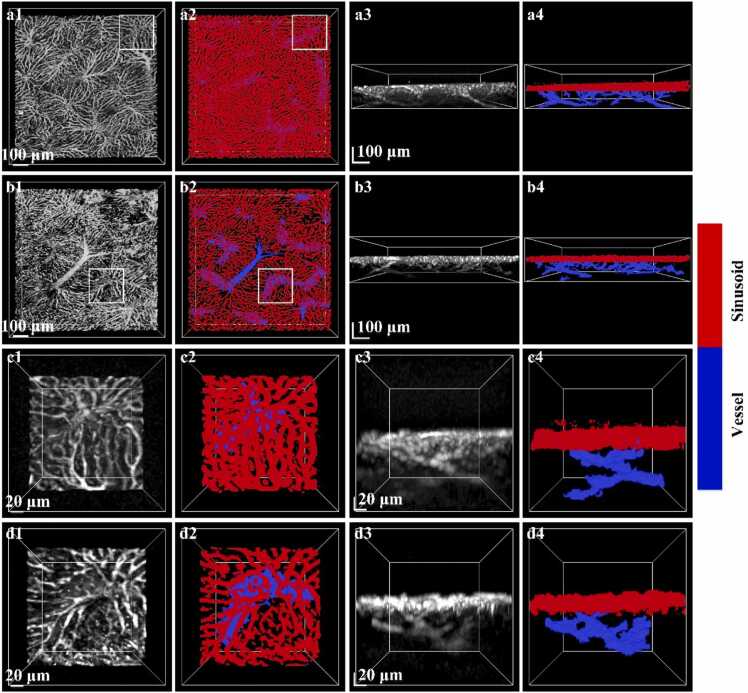
3D images hepatic sinusoids and vessels pre- and post-separation 3d images of normal and early nash mouse: (a1–a2) Pre- and post- separation 3D images of hepatic sinusoids and vessels in the normal mouse liver (XY plane). (a3-a4) corresponding views of (a1-a2) in the XZ plane. (b1-b2) Pre- and post- separation 3D images of hepatic sinusoids and vessels in early NASH mouse liver (XY plane). (b3-b4) corresponding views of (b1-b2) in the XZ plane. (c1-c2) Magnified views of the White-boxed regions in (a1) and (a2). (c3-c4) corresponding views of (c1-c2) in the XZ plane. (d1-d3) Magnified views of the White-boxed regions in (b1) and (b2). (d3-d4) further magnified views of White-boxed regions in (d1) and (d2).

We utilized the LIM system to acquire microvascular images of mouse livers and applied the proposed method for separation of vessels and hepatic sinusoids. To quantitatively evaluate the separation performance, adaptive thresholding was first applied to the original image to generate a binarized result that maximally preserves structural information. This result was used as a reference in the unseparated state for subsequent quantitative comparison with the separation outcome. In all separated images, red represents hepatic sinusoids, while blue denotes vessels. Fig. 7 illustrates the 3D images of hepatic sinusoids and vessels before and after separation in both normal and early-stage NASH mice.

Specifically, Fig. 7(a1) and Fig. 7(a2) display pre- and post-separation images in the XY plane for the normal group, while Fig. 7(a3) and Fig. 7(a4) show the corresponding XZ views. Similarly, Fig. 7(b1) and Fig. 7(b2) illustrate the XY plane images for the early-stage NASH group, and Fig. 7(b3) and Fig. 7(b4) show their XZ counterparts. The FOV for all images is 1.5 mm × 1.5 mm × 0.3 mm. As revealed by the comparative analysis, after separation, weak signals were enhanced, noise around vessels was reduced, and scattered artifacts between sinusoids were eliminated, enabling clearer visualization of the spatial organization of liver vasculature. In early-stage NASH mice, originally disordered vascular regions became well-defined microvascular structures, thereby improving the clarity and hierarchy of the liver microstructure. To further validate the separation performance, the white-boxed regions in Fig. 7(a1), 7(a2), 7(b1), and 7(b2) (field of view: 0.3 mm × 0.3 mm × 0.3 mm) were magnified. The corresponding magnified results are presented in Fig. 7(c1)–(c4) and Fig. 7(d1)–(d4), which were derived from the normal and NASH groups, respectively, and sequentially display the pre- and post-separation XY and XZ views. These enlarged images demonstrate a clear enhancement in SNR, with improved Z-axis distribution and removal of artifacts and false connections.

For quantitative analysis, three mice were selected, and three parameters (i.e., sinusoid radius, tortuosity, and density) were calculated before and after the separation of hepatic sinusoid and vessel images. All quantitative results are summarized in Table 2. In the healthy group, after separation, sinusoid density and tortuosity decreased by 1.6 % ± 0.001 and 2.8 % ± 0.011, respectively, while the average sinusoid radius increased by 41.95 % ± 0.318. In the early disease group, density and tortuosity decreased by 46.48 % ± 0.04 and 12.38 % ± 0.018, respectively, with a corresponding 5.68 % ± 0.011 increase in average radius. Furthermore, when comparing the healthy and early disease stages, prior to separation, sinusoid density, tortuosity, and average radius increased by 4.42 % ± 0.073, 2.50 % ± 0.023, and 2.03 % ± 0.018, respectively. After separation, these values decreased by 43.06 % ± 0.019, 8.43 % ± 0.020, and 17.37 % ± 0.017, respectively.

**Table 2:** Quantitative analysis of hepatic sinusoids and vessels pre- and post- separation.

		**Mouse-Normal**			**Mouse-Early**		
		**Density**	**Radius**	**Tortuosity**	**Density**	**Radius**	**Tortuosity**
Mouse1	**Unsep**	0.0956	2.4781	0.5573	0.1078	2.5064	0.5519
	**Sep**	0.0941	3.2731	0.5375	0.0552	2.6495	0.4822
Mouse2	**Unsep**	0.0961	2.5345	0.5495	0.0931	2.5129	0.5791
	**Sep**	0.0945	3.2699	0.5313	0.0541	2.6995	0.4978
Mouse3	**Unsep**	0.0953	2.4063	0.5534	0.1005	2.5097	0.5455
	**Sep**	0.0938	3.1215	0.5349	0.0515	2.6345	0.4884

Significant differences in sinusoidal density, diameter, and tortuosity were observed between pre- and post-separation images. These discrepancies are primarily due to the complex overlap of vessels and sinusoids in unseparated images, which obscures their boundaries. Since vessels generally exhibit higher density, larger diameters, and lower tortuosity than sinusoids, failure to accurately separate them results in parameter cross-contamination, distorting quantitative outcomes. Thus, precise separation of hepatic sinusoids and vessels is essential for accurate quantification. It not only improves the resolution of sinusoidal structures but also ensures reliable measurement of parameters.

### 3.4 Dynamic changes in liver microvascular structures during NASH progression

**Fig. 8:**
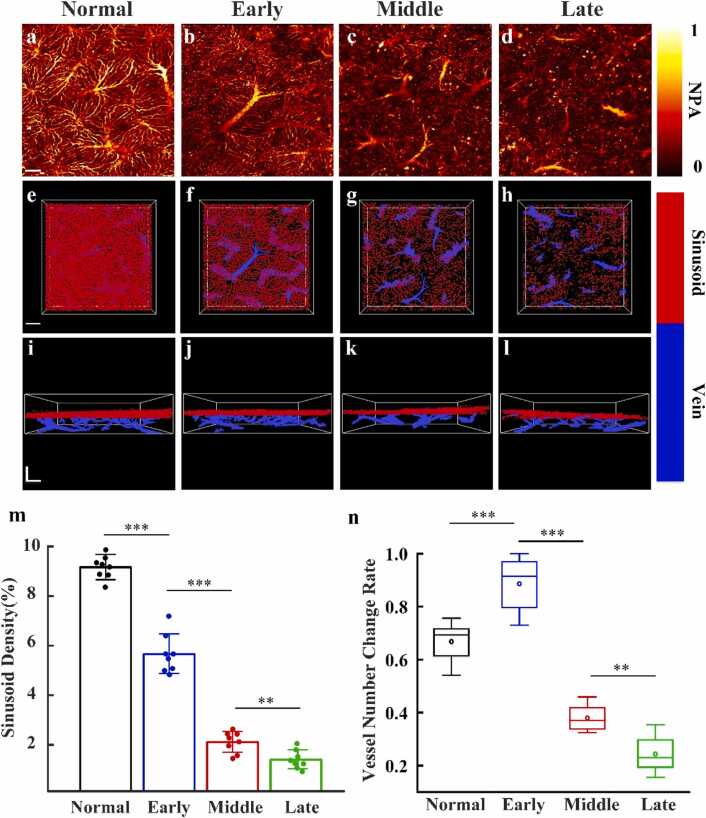
PAM images of mouse liver at healthy and different disease stages**.** (a-d) 3D *p*AM images of microcirculation in the mouse liver. (e-h) 3D separation images of hepatic sinusoids and vessels in the XY *p*lane. (I-l) 3D separation images of hepatic sinusoids and vessels in the XZ *p*lane. (m). Quantitative statistics of hepatic sinusoid density. N. quantitative statistics of vessel count. All scale bars: 100 µm. ns indicates no significant difference, * *p* < 0.05, ** *p* < 0.01, *** *p* < 0.001, **** *p* < 0.0001.

Fig. 8(a)–(d) shows photoacoustic MAPs of hepatic microvascular structures in the normal group and early, mid, and late stages of NASH. The normal liver (Fig. 8(a)) is characterized by well-organized hepatic sinusoids and a continuous, dense microvascular network. In early-stage NASH (Fig. 8(b)), irregularities begin to emerge in the microvascular network, along with a slight reduction in sinusoid density. Despite these changes, the overall architecture remains largely intact, and vascular continuity is preserved. By the mid-stage (Fig. 8(c)), the microvascular structures become increasingly blurred, with notable signal loss and the formation of localized vascular nodules. Inflammatory responses triggered by a high-fat diet, along with lipid accumulation, contribute to the compression and destruction of microvascular structures. Concurrently, hemoglobin concentration decreases significantly, limiting excitation of microvascular signals at the 532 nm wavelength. In late-stage NASH (Fig. 8(d)), the microvascular network exhibits severe deterioration, vessels become twisted, constricted, occluded, or entirely absent, accompanied by extensive signal loss and widespread vascular nodules. These changes result in substantial disruption of hepatic microcirculation.

Fig. 8(e)-(h) presents 3D images of the liver microvascular network in the XY plane after separation of hepatic sinusoids and vessels for the normal group and the early, mid, and late stages of NASH. Corresponding XZ plane views are shown in Fig. 8(i)-(l), with vessels depicted in blue and hepatic sinusoids in red. In the normal liver (Fig. 8(e)), the dense hepatic sinusoid network nearly obscures vessels in the XY view. However, the XZ view (Fig. 8(i)) reveals vessels extending upward from the base and branching into hepatic sinusoids, indicating strong network continuity and integrity. In early-stage NASH (Fig. 8(f) and (j)), hepatic sinusoid density begins to decline, exposing more of the underlying vascular structure. Nonetheless, the overall architecture and vascular continuity remain comparable to the normal group. Mild lipid accumulation leads to slight attenuation of sinusoid signals, while the bottom vessels remain unaffected and signal intensity remains relatively stable.

In mid-stage NASH (Fig. 8(g) and (k)), increased lipid accumulation and inflammation severely compromise network integrity. Hepatic sinusoid density drops significantly, continuity is disrupted, and localized damage and nodule formation become evident. The number of bottom vessels also decreases, indicating substantial network degradation. By late-stage NASH (Fig. 8(h) and (l)), the hepatic microvascular network is profoundly damaged. Both sinusoids and vessels exhibit widespread rupture and fragmentation, with some regions entirely lost. As scar tissue forms, the liver transitions to cirrhosis. This extensive damage signifies irreversible breakdown of the hepatic microcirculatory network in advanced disease stages.

Then, a quantitative analysis of hepatic sinusoid density and vessel count ratio was conducted across different stages of disease progression. The method for calculating hepatic sinusoid density is described in Section 2.3, while the vessel count ratio was normalized using the maximum value across all datasets as a reference. As shown in Fig. 8(m) and Fig. 8(n), healthy mice exhibited a hepatic sinusoid density of 0.091 ± 0.0047 and a vessel count ratio of 0.67 ± 0.07, representing the normal microcirculatory structure of the liver. In early-stage NASH, sinusoid density significantly declined to 0.056 ± 0.008, while the vessel count ratio increased to 0.89 ± 0.10. These changes suggest that early pathological alterations primarily affect the sinusoidal layer, with deeper vascular structures remaining relatively intact. Lipid accumulation leads to the fragmentation and loss of superficial sinusoids, enhancing PA signal excitation from deeper vessels and making them more prominent in imaging.

In the mid-stage of NASH, sinusoid density further declined to 0.02 ± 0.004, and the vessel count ratio dropped sharply to 0.38 ± 0.04. This indicates substantial damage to the microcirculatory network due to lipid accumulation and inflammation, disrupting the continuity of both vessels and hepatic sinusoids. By the late stage, sinusoid density decreased to 0.012 ± 0.004, and the vessel count ratio fell to 0.24 ± 0.06, reflecting severe microvascular destruction, with widespread loss of vessels and sinusoids and near-total failure of microcirculation.

These findings reveal the dynamic deterioration of hepatic sinusoid density and vessel count ratio throughout NASH progression. In the early stage, pathological changes mainly affect hepatic sinusoids, while vessels remain largely preserved. By the mid-stage, lipid accumulation and inflammatory cell infiltration begin to impair vascular structures, significantly compromising both sinusoid and vessel integrity. In the mid-to-late stages, the microvascular network exhibits near-complete loss of structure and function, accompanied by extensive hepatic scarring. These results underscore the profound impact of lipid accumulation and inflammation on the liver’s microvascular architecture.

### 3.5 Quantitative analysis of the spatial distribution between sinusoids and vessels

**Fig. 9:**
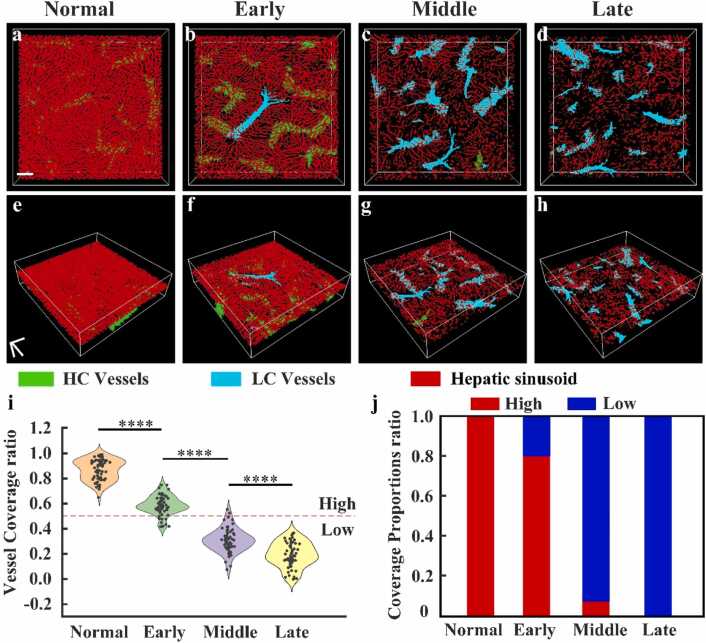
Vascular coverage in mouse liver across health and various disease stages: (a-d) 3D *p*AM images of vascular coverage classification in XY *p*lane for mouse livers at different stages (normal, early, mid, and late NASH). (d-h) 3D *p*AM images of vascular coverage classification in the XYZ *p*lane for mouse livers at different stages (normal, early, mid, and late NASH). (m) quantitative statistics of vascular coverage. (n) *p*roportion statistics of vessels with different coverage rates. All scale bars: 100 µm. **** *p* < 0.0001.

To better characterize the spatial distribution characteristics of vessels and hepatic sinusoids during NASH disease progression, vessels were classified into high-coverage (HC) and low-coverage (LC) categories based on the extent to which hepatic sinusoids envelop the vessel surface. The spatial relationship between hepatic sinusoids and vessels was quantified by measuring the coverage rate of hepatic sinusoids over vessels, as described in Section 2.3. Vessels with a coverage rate > 50 % were classified as high-coverage, while those with < 50 % were classified as low-coverage. HC vessels are extensively covered by sinusoids, forming a dense microvascular network that may facilitate more efficient substance exchange and blood flow regulation. In contrast, LC vessels are surrounded by sparsely distributed sinusoids, resulting in reduced vessel–sinusoid contact and potentially weaker microcirculatory function. The corresponding 3D visualizations in the XYZ plane are shown in Fig. 9(e-h), where green indicates high-coverage vessels, cyan indicates low-coverage vessels, and red represents hepatic sinusoids.

Fig. 9(i) displays the distribution of vessel coverage across groups, with each point representing a vessel. The red dotted line (coverage rate = 0.5) separates high- from low-coverage vessels. In healthy mice, the mean vascular coverage rate is 0.871 ± 0.081. This declines to 0.704 ± 0.098 in early-stage NASH, 0.313 ± 0.092 in mid-stage, and 0.197 ± 0.098 in the late stage. Most vessels in the healthy and early-stage groups are classified as high-coverage, while mid- and late-stage groups show a clear shift toward low-coverage, indicating a sharp transition in the spatial relationship between sinusoids and vessels during disease progression.

To further assess these changes, a statistical analysis of the proportions of high- and low-coverage vessels was conducted, as shown in Fig. 9(j). In the normal group, nearly all vessels are high-coverage, indicating that hepatic sinusoids almost completely envelop the vascular structures, supporting the integrity of the liver microcirculatory network. In early-stage NASH, high-coverage vessels drop by 20 %, while low-coverage vessels increase by 80 %, reflecting reduced sinusoidal density in vascular regions and decreased red blood cell velocity. In the mid-stage, high-coverage vessels decline sharply by 7 %, while low-coverage vessels rise by 93 %, indicating severe damage to sinusoid–vessel connectivity due to lipid accumulation and inflammation. By the late stage, high-coverage vessels are nearly absent, and low-coverage vessels account for 100 % of the observed structures. At this point, microcirculatory function is irreversibly compromised, with severe ischemia and disrupted material exchange caused by extensive scar tissue.

### 3.6 Quantitative analysis of hepatic sinusoids

**Fig. 10:**
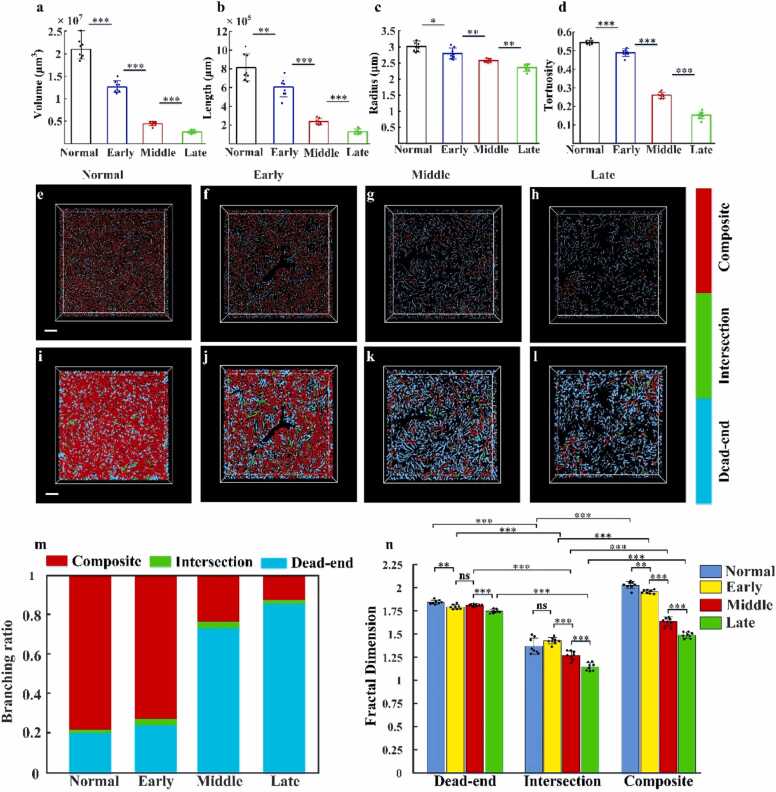
Comparison of morphological *p*arameters of hepatic sinusoids in healthy and different stages of disease: **(**a) blood volume of hepatic sinusoids in health and different stages of disease. (b) Length of hepatic sinusoids in health and different stages of disease. (c) Radius of hepatic sinusoids in health and different stages of disease. (d) Tortuosity of hepatic sinusoids in health and different stages of disease. (e-h) Skeleton branching classification results of hepatic sinusoids in health and different stages of disease. (I-l) Branching classification results of hepatic sinusoids in health and different stages of disease. (m) *p*ercentage statistics of different categories of hepatic sinusoid branches in health and different stages of disease. (n) Fd of different categories of hepatic sinusoid branches in health and different stages of disease. All scale bars: 100 µm. ns indicates no significant difference, * *p* < 0.05, ** *p* < 0.01, *** *p* < 0.001, and **** *p* < 0.0001.

Changes in the dynamic equilibrium of the sinusoidal structure are a key factor in the progression of NASH. To assess these alterations, changes in key structural parameters (i.e., sinusoid length, volume, radius, and tortuosity) were quantified and compared across disease stages. As shown in Fig. 10(a-d), hepatic sinusoids exhibit significant reductions in all parameters from the healthy state to the early and late stages of NASH. In healthy mice, the sinusoidal volume is (2.11 ± 0.08) × 10^7^ μm^3^, the length is (8.12 ± 0.4) × 10^5^ μm, the radius is 3.05 ± 0.05 μm, and the tortuosity is 0.54 ± 0.003. In the early stage, these values decrease to (1.28 ± 0.04) × 10^7^ μm^3^, (6.06 ± 0.37) × 10^5^ μm, 2.79 ± 0.06 μm, and 0.48 ± 0.006, respectively. From the early to mid-stage, there is a sharp decline: volume drops to (4.5 ± 0.16) × 10^6^ μm^3^, length to (2.37 ± 0.11) × 10^5^ μm, radius to 1.31 ± 0.09 μm, and tortuosity to 0.26 ± 0.005. In the late stage, the values further decrease to (2.6 ± 0.12) × 10^6^ μm^3^, (1.31 ± 0.09) × 10^5^ μm, 2.38 ± 0.03 μm, and 0.15 ± 0.007, respectively.

Based on these morphological changes, graph theory was applied to analyze sinusoidal branching structures, and branch classifications were visualized. Quantitative analysis of branch types and their corresponding fractal dimensions was performed, with ratios normalized to the maximum observed value. Full methodology is provided in Section 2.3. Fig. 10(e-h) shows the skeletal branch classification for each disease stage, while Fig. 10(i-l) presents the corresponding visualized results. Fig. 10(m) summarizes the proportions of complex (red), crossing (green), and dead-end (cyan) branches. In healthy mice, complex branches account for 78 %, crossing for 1 %, and dead-end for 21 %. As the disease progresses, complex branches decline while dead-end branches rise. From the early to mid-stage, complex branches drop from 73 % to 24 %, and dead-end branches increase from 25 % to 74 %; crossing branches remain relatively stable (∼2 %). In the late stage, the trend continues: complex branches fall to 13 %, dead-end branches rise to 86 %, and crossing branches slightly decrease to 1 %.

Finally, Fig. 10(n) presents a statistical comparison of the FDs for the three branch types across all disease stages (i.e., early, mid, and late). The calculation of FD is described in Section 2.3. Among the three branch types, composite branches exhibited the most pronounced changes, with FD values declining progressively from 2.028 ± 0.014 in healthy tissue to 1.958 ± 0.007 (early), 1.636 ± 0.017 (mid), and 1.488 ± 0.011 (late), corresponding to a substantial 26.62 % reduction throughout disease progression. Intersection branches initially increasing from 1.369 ± 0.030 (healthy) to 1.427 ± 0.012 (early) before undergoing progressive deterioration to 1.272 ± 0.017 (mid) and 1.147 ± 0.015 (late). This resulted in an overall decline of 16.2 % from the healthy baseline to the late stage. In contrast, dead-end branches showed the most modest alterations, with FD values gradually decreasing from 1.845 ± 0.008 (healthy) through 1.793 ± 0.008 (early) and 1.783 ± 0.004 (mid) to 1.758 ± 0.007 (late), representing a relatively small but consistent 4.7 % reduction over the entire disease course.

Data analysis indicates that composite branches exhibited the most pronounced changes, corresponding to a substantial 26.62 % reduction throughout disease progression. Intersection branches also showed a significant decline of 16.2 % from the healthy baseline to the late stage. In contrast, dead-end branches demonstrated the most modest alterations, with a relatively small but consistent 4.7 % reduction over the entire disease course. These findings suggest a marked decrease in the structural complexity of hepatic sinusoids, potentially indicating severe disruption of sinusoidal network connectivity and a progressive deterioration of hepatic microcirculatory function.

Collectively, these results reveal that as NASH progresses, hepatic sinusoids undergo substantial morphological deformation. The increase in sinusoidal pressure likely reduces blood flow, leading to local hypoxia, which in turn promotes lipid peroxidation and inflammation. This cascade ultimately disrupts the organization of the liver microvascular architecture.

### 3.7 Histopathological examination

**Fig. 11:**
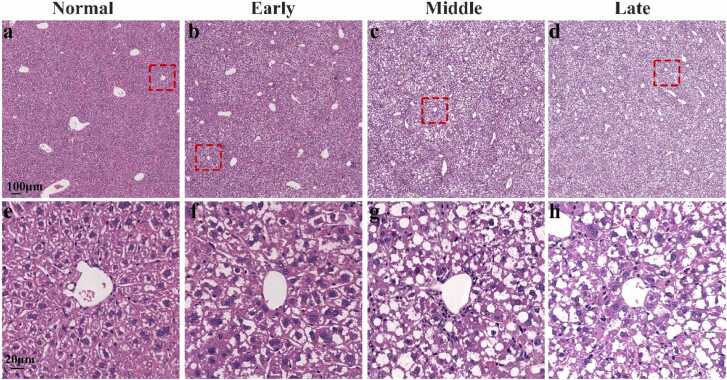
Histopathological sections of mouse livers were stained with h&e: (a-d) H&E images of liver tissues from the normal, early, middle, and late stages. (e-h) High-magnification views of red dashed box areas in (a-d), respectively, highlighting progressive microstructural alterations within hepatic lobules.

Fig. 11 presents H&E-stained histological images showing hepatic tissue changes at different stages of NASH progression. In the normal group (Fig. 11(a)), the liver tissue exhibits clear structure and relatively intact sinusoidal distribution, and no obvious lipid droplets are observed. Fig. 11(e) shows a magnified representative region from Fig. 11(a). In the early stage (Fig. 11(b)), some hepatocytes appear swollen, and small lipid droplets may be present, suggesting the possibility of early steatosis. Fig. 11(f) shows a magnified representative region from Fig. 11(b). In the middle stage (Fig. 11(c)), hepatocyte swelling seems to worsen, and lipid droplets appear to increase. Based on previous studies [30], [31], the presence of lipid vacuolation may be inferred. Fig. 11(g) shows a magnified representative region from Fig. 11(c). In the late stage (Fig. 11(d)), steatosis appears to worsen further, with what seems to be a large accumulation of lipid droplets. Sinusoidal appear unclear in some regions, indicating structural disruption. Fig. 11(h) shows a magnified representative region from Fig. 11(d).

## 4 Discussion and conclusion

This study developed a semi-invasive, label-free imaging and analysis method that, in combination with MLW technology, enables long-term visualization of liver microvasculature in both normal and NASH conditions for the first time. Utilizing LPAM technology, this method allows for high-resolution, longitudinal monitoring and analysis of hepatic microvascular structural changes throughout disease progression. PAM images were successfully separated into two primary structures: vessels and hepatic sinusoids. Quantitative analysis revealed that both structures undergo significant alterations as NASH progresses. Moreover, spatial distribution analysis indicated that the connectivity and integrity of the hepatic microcirculatory network are severely disrupted, leading to compromised liver function.

The integration of OR-PAM with MLW and LIM provides a robust technological platform for tracking microvascular changes in the liver over time. Compared to conventional drawer-type liver windows, our setup minimizes vibration-related interference, resulting in enhanced imaging stability and quality. The LIM system is cost-effective and straightforward to assemble using 3D-printed or commercially available components. Additionally, the use of Ut-PMMA as the window material improves optical clarity and signal reception. This approach also supports compatibility with other imaging modalities, such as micro-ultrasound and fluorescence microscopy, increasing its adaptability and potential applications. Crucially, our method avoids the need for fully invasive procedures, thereby enabling dynamic, long-term studies of NASH-related liver microvascular remodeling and providing a reliable imaging basis for subsequent quantitative analysis.

The liver microvasculature comprises two distinct structures: vessels with intact vascular walls (e.g., portal veins) and hepatic sinusoids, which consist of fenestrated endothelial cells that facilitate exchange of nutrients and waste products. Despite their functional importance, no prior study has focused on the separation of hepatic sinusoids and vessels in PAM images. Our study addresses this gap through 3D quantitative analysis of both structures across NASH stages, highlighting PAM’s unique advantage in liver disease research. One key technical challenge in hepatic sinusoid and vessel separation is the attenuation of PA signals from deeper vasculature, which are often obscured by signal absorption in overlying sinusoids. To overcome this, vessels-sinusoids separation and enhancement method was developed. First, an image enhancement technique based on percentile analysis and histogram matching was applied to mitigate depth-related signal attenuation. Then, using the typical diameter of hepatic sinusoids [23], [24], [25], [26] as reference, we set the structural element radius for erosion operations (r = 4 μm) to remove sinusoidal structures, and obtained separated hepatic sinusoid data by subtracting the separated vessel data from the original enhanced images. Finally, hessian matrix filtering [32] and adaptive thresholding [27] were applied to obtain binary images. This method effectively suppressed background noise, enhanced signal fidelity, and provided a foundation for precise three-dimensional reconstruction of hepatic microvascular structures.

Quantitative comparisons further confirmed the value of our vessel and hepatic sinusoid separation method: the density and tortuosity of hepatic sinusoids were reduced by 22.51 % and 6.51 %, respectively, while the average sinusoidal diameter increased by 20.25 % compared to unseparated data. Subsequently, a multi-parameter quantitative analysis was conducted to comprehensively characterize microvascular changes across different stages of NASH. These indicators provide a robust foundation for elucidating pathological alterations in liver microvasculature and their association with microcirculatory dysfunction during disease progression.

In addition, the spatial distribution of vessels and hepatic sinusoids was analyzed by introducing a novel metric, sinusoidal coverage of vessels, to classify vessels into high- and low-coverage categories. This classification integrates voxel-based spatial statistics, and Z-axis coverage range to quantitatively depict the spatial organization of the hepatic microcirculatory network. By advancing beyond traditional MAP-based image reconstruction, our method enables more accurate 3D spatial characterization, thereby addressing previous limitations and expanding the application of PA microscopy in liver microcirculation research.

Quantitative results revealed a consistent decline in key structural parameters (including the number of vessels and the radius, volume, length, tortuosity, and density of hepatic sinusoids) as NASH progressed. These changes were particularly pronounced from the early to middle stage, with a slower rate of deterioration observed in the late stage. Regarding microvascular complexity and network patterns, disease progression led to a reduction in sinusoid density, a marked decrease in complex branches, an increase in dead-end branches, and a decline in the fractal dimensions of all branch types. Spatial coverage analysis further revealed a significant reduction in the degree to which hepatic sinusoids envelop vessels, indicating progressive disruption of sinusoid-vessel structural association.

This phenomenon is more likely the result of multiple pathological factors acting in combination, including intracellular lipid accumulation, narrowing of the perisinusoidal space, and collagen deposition within the sinusoidal lumen—all of which can impair blood flow within hepatic sinusoids. At the same time, lipid accumulation and deposition further exacerbate hepatic steatosis. Lipids released by necrotic hepatocytes can form emboli that obstruct sinusoidal channels and reduce local hemoglobin concentrations [33]. The resulting decrease in hemoglobin content leads to reduced optical absorption, ultimately manifesting as signal loss in photoacoustic imaging. Coverage-based spatial analysis of the relationship between hepatic sinusoids and vessels further supports previous findings [34], which suggested a reduction in the number of hepatic sinusoids within vessels regions and a decrease in red blood cell velocity in NASH mouse models. To further substantiate our imaging observations—particularly the apparent reduction of HC vessels in the late stage. H&E-stained ex vivo tissue sections were incorporated to evaluate morphological changes in hepatic microstructures at different stages of NASH (Section 3.7 and Fig. 11). These images show that, especially in the late stage, the sinusoidal structures surrounding the vessels in certain regions appear to exhibit a degree of disorganization and potential disruption. In some areas, signs of structural blurring or partial loss can be observed, suggesting that the structures may have sustained a certain degree of damage.

Despite its strengths, the LAPM system has several limitations that warrant consideration. Firstly, although the MLW & LIM setup reduces animal movement through mechanical stabilization, it does not actively compensate for organ displacement caused by respiration. The observed reduction in motion artifacts mainly results from sedation and fixation, rather than active motion correction. Secondly, the current XYZ mechanical scanner limits imaging speed, efficiency, and field of view, making it difficult to revisit the same hepatic region across disease stages. High-speed scanning methods like galvanometers or microelectromechanical systems (MEMS) [35], [36], could enhance temporal-spatial consistency for accurate longitudinal studies. Additionally, high absorption of light in the surface of liver results in hepatic sinusoids in deeper tissue layers exhibiting extremely low SNR. Therefore, they are difficult to be visualized in the segmentation results. Notably, improving the spatial resolution of PAM systems [37], [38], [39] enables in-depth analysis of cellular-level microcirculation [40], [41], [42], which is critical for understanding the pathogenesis of NASH. At last, it should be emphasized that, referencing the hepatic sinusoidal diameters obtained from multiple high-resolution imaging systems [23], [24], [25], [26], the radius of the structural element used for erosion in this study was set to 4 μm for hepatic sinusoids. Additionally, H&E-stained ex vivo tissue sections were incorporated to evaluate morphological changes in hepatic microstructures at different stages of NASH (Section 3.7 and Fig. 11). The results indicate that there may be diameter differences between sinusoids and vessels. Under pathological conditions, the size-based method for separating hepatic sinusoids and vessels may have certain limitations. Inflammatory responses and steatosis can alter vascular morphology, and some small vessels may resemble sinusoids in diameter, thereby increasing the risk of incorrect separation, leading to a local overestimation of sinusoid-related parameters. However, this does not unduly compromise the overall robustness of the separation strategy and the main conclusions of the study. Furthermore, given the differences in oxygen saturation and blood flow velocity between vessels and hepatic sinusoids under both normal and diseased states [43], functional photoacoustic imaging [44], [45] holds promise as a complementary tool to further improve separation accuracy.

LPAM does not require exogenous contrast agents, enabling researchers to assess the effects of various compounds on the hepatic microcirculatory system without disturbing the physiological environment of mice, while preserving the integrity of subsequent histological analyses. Additionally, this technique supports localized drug delivery and injection, thereby facilitating experimental therapeutic research targeting NASH. The approach is also applicable to imaging other abdominal organs, such as the kidneys. Overall, LPAM offers an innovative strategy for the longitudinal investigation of NASH and holds broad potential for biomedical imaging applications.

## Funding

This work was supported by the 10.13039/501100001809National Natural Science Foundation of China (32330048), the Science and Technology Talent Innovation Project in Hainan Province (KJRC2023A03, KJRC2023B09), and the Innovation Fund of WNLO.

## CRediT authorship contribution statement

**Xiaoquan Yang:** Writing – review & editing, Supervision, Funding acquisition. **Qingming Luo:** Resources, Funding acquisition. **Zhihong Zhang:** Supervision, Resources, Funding acquisition. **Yanfeng Dai:** Writing – review & editing. **Lulu Gao:** Investigation. **Jianshuang Wei:** Writing – original draft, Investigation, Formal analysis, Data curation. **Liu Xiuli:** Supervision. **Ren Zhang:** Writing – original draft, Visualization, Investigation, Formal analysis, Data curation. **Mingchen Jiang:** Data curation. **Ximiao Yu:** Formal analysis.

## Declaration of Competing Interest

The authors declare no conflict of interest exists.

## Data Availability

Data will be made available on request.
